# A retrospective cohort study and spatial analysis of climate and community-level determinants of respiratory syncytial virus notifications among Queensland infants, prior to the introduction of the RSV mother and infant protection program (RSV-MIPP) immunisation initiative

**DOI:** 10.1186/s12889-026-26288-6

**Published:** 2026-01-22

**Authors:** Sarah Graham, Benn Sartorius, Tom Snelling, Nusrat Homaira, Anthony T. Newall, Michael Binks, Colleen Lau, Catherine Hughes, Lisa McHugh

**Affiliations:** 1 School of Public Health, Faculty of Medicine, University of Queensland, Level 0, Room 011, Bldg 887, 288 Herston Road, Brisbane, QLD 4006 Australia; 2 Centre for Clinical Research, Faculty of Medicine, University of Queensland, Brisbane, Australia; 3 Sydney School of Public Health, Faculty of Medicine and Health, University of Sydney, Sydney, Australia; 4 Discipline of Paediatrics and Child Health, Faculty of Medicine and Health, University of New South Wales, Sydney, Australia; 5 Respiratory Department, Sydney Children’s Hospital, Sydney, Australia; 6 School of Population Health, Faculty of Medicine and Health, University of New South Wales, Sydney, Australia; 7 Maternal and Child Health Division, Menzies School of Health Research, Charles Darwin University, Darwin, Australia; 8 Women and Kids Theme, South Australian Health and Medical Research Institute, Adelaide, Australia; 9 Consumer/Community Representative, Immunisation Foundation of Australia, Sydney, Australia

**Keywords:** Immunisation, Vaccination, Maternal, Child, Infant, RSV, Queensland

## Abstract

**Background:**

Respiratory syncytial virus (RSV) is a highly infectious seasonal respiratory pathogen and a major cause of morbidity in young children. In Australia, RSV is the leading cause of hospitalisation for bronchiolitis and pneumonia among children aged < 2 years, with the highest severity observed in infants aged < 6 months. RSV became a nationally notifiable condition in July 2021, and the national RSV Mother and Infant Protection Program (RSV-MIPP) commenced in February 2024. Baseline data on RSV incidence and its determinants are needed to evaluate the effectiveness of the program and identify populations at greatest risk.

**Methods:**

Retrospective cohort study with spatial analysis of all RSV notifications among children aged < 2 years residing in the state of Queensland, Australia between 1 January 2022 and 31 December 2023. Data were obtained from the Queensland Notifiable Conditions System. Incidence rates were calculated by exact age in infant months, year, epidemiological week, and climate zone, using all resident children as the denominator population. Spatial cluster analysis methods identified postcode areas with high incidence, and associations in climate zones, and community-level characteristics (remoteness, socioeconomic status, average number of children per family household).

**Results:**

18,683 notifications were recorded among children aged < 2 years between 2022−2023 (79.7 per 1,000 in 2022; 84.8 per 1,000 in 2023). Incidence was consistently higher among 1-month-olds (96.6 per 1,000) and 12-month-olds (96.7 per 1,000). Compared to tropical climates, incidence was higher in temperate (aRR 1.26, 95% CI 1.13−1.41) and arid/semi-arid zones (aRR 1.18, 95% CI 1.00−1.38), with differences in timing and magnitude of epidemics between climate zones. Higher incidence was observed in areas with larger family sizes (aRR 1.39, 95% CI 1.13−1.72). Remoteness was associated with lower incidence (aRR 0.89, 95% CI 0.87−0.92).

**Conclusions:**

In Queensland, children living in areas with larger family sizes and temperate or arid/semi-arid climates experienced higher incidence of RSV infections. Lower recorded incidence in remote areas may reflect undertesting or lower-case ascertainment. Future RSV-MIPP strategies should prioritise climatic and community-level determinants through targeted outreach and enhanced surveillance to facilitate equitable access. There is an urgent need for new strategies to protect infants aged > 6 months, when protection from maternal vaccination and birth dose therapeutics wanes.

**Supplementary Information:**

The online version contains supplementary material available at 10.1186/s12889-026-26288-6.

## Background

Respiratory syncytial virus (RSV) is a highly infectious seasonal respiratory disease and is a significant cause of morbidity among young children [1]. Globally, RSV is estimated to cause approximately 20 million lower respiratory tract infections and over 2 million hospitalisations each year among children < 2 years [1]. It is the leading cause of hospitalisation for bronchiolitis and pneumonia among Australian children aged < 2 years, with greatest severity observed in early infancy [1, 2]. RSV is estimated to cause 12,000–15,000 hospital admissions annually in Australia, with associated healthcare costs of approximately AUD 59–121 million per year [3]. Approximately half of all paediatric RSV-related hospital and Intensive Care Unit (ICU) admissions in Australia are among infants < 6 months of age [2]. The burden is disproportionately higher among First Nations infants, who experience substantially higher rates of RSV infections and hospitalisations [2]. Birth shortly before or during RSV season, household crowding, living with siblings, daycare attendance and socioeconomic disadvantage are frequently cited as factors associated with higher risk of RSV infections [4–6].

Timing of seasonal RSV epidemics varies between Australia’s climate zones [7, 8]. In temperate regions, RSV activity typically begins in April to May, and peaks during the southern hemisphere winter months of June to August [8, 9]. Tropical areas may experience year-round circulation or monsoonal peaks [7, 8], however arid zones remain poorly characterised. Climate change is expected to alter regional epidemic dynamics over time [10].

In 2023−2024, the Australian Government Therapeutic Goods Administration (TGA) approved two novel preventative agents to reduce RSV incidence and severity in infants aged < 6 months [11, 12]. These include a maternal RSV vaccine offered during the third trimester of pregnancy (Abrysvo), and long-acting RSV-specific monoclonal antibodies for infants (nirsevimab) [13]. Since 01 February 2024, the health department in the Australian state of Queensland (Queensland Health) has provided year-round access to a free birth-dose of nirsevimab for all infants. This is available up until 8 months of age, or < 2 years in children with additional risk factors for severe RSV disease [14]. All Australian states and territories now provide similar programs with varying eligibility requirements, and year-round or seasonal availability [13, 15]. This variation reflects the current national landscape with Queensland, Northern Territory, and north of Western Australia offering nirsevimab year-round, while Australia’s southernmost states and territories where winters are cooler restrict access to the seasonal RSV period (April–September/October) [16]. Jurisdictional differences highlight the dynamic and evolving nature of RSV prevention policy across Australia. Jurisdictional nirsevimab programs form part of the national RSV Mother and Infant Protection Program (RSV-MIPP), which since February 2025 has additionally provided free access to RSV vaccination for all pregnant women under the National Immunisation Program (NIP) [13, 15].

RSV was designated a nationally notifiable condition in July 2021 [17]. With nirsevimab introduced in Queensland in February 2024, the years 2022−2023 represent the only complete calendar years of RSV notification data available prior to the introduction of RSV immunisations. Queensland is geographically large, covering multiple climate zones. However to our knowledge, no population-level analysis has quantified community or climatic determinants of RSV incidence among Queensland infants prior to the RSV-MIPP. Understanding the epidemiology, community-level determinants, and spatiotemporal dynamics of RSV incidence among Queensland infants prior to the introduction of the RSV-MIPP is critical for evaluation of these funded programs, and for achieving optimal and equitable state-wide coverage.

The objectives of this study were to quantify pre-program incidence of RSV notifications among Queensland’s children aged < 2 years, describe spatiotemporal differences in this incidence between Queensland’s diverse climate zones, and to identify high-incidence spatial clusters and community-level determinants of incidence. We hypothesised that RSV incidence and seasonality would vary by climate zone, that incidence patterns in arid and semi-arid regions where epidemiological data are limited would be characterised for the first time, and that higher rates would occur in areas with greater household crowding and population density.

## Methods

### Setting

Queensland is a geographically large state in north-eastern Australia, covering approximately 1.73 million km^2^. The state features significant climate zone diversity, including coastal areas of consistently high humidity and rainfall, a southern inland temperate region, and semi-arid and arid regions toward the state’s interior [18]. Queensland’s population of approximately 5.6 million is concentrated in cities and towns along the eastern coastline, with regional and remote populations dispersed throughout the northern tropical coast and the expansive dry interior [18, 19]. Around 4.6% of Queensland’s population identifies as Aboriginal and/or Torres Strait Islander [20], representing the second largest First Nations population of any Australian state or territory [21]. Birth rates among First Nations peoples remain higher than the state average, contributing to a relatively younger age structure [21]. Queensland’s annual population growth rate of 2.0% is slightly above Australia’s national average 1.8%, with 58,827 births recorded in the year between 30 September 2023−2024 [19].

## Study design and inclusion criteria

For this retrospective cohort study with spatial analysis, we included all children aged < 2yrs residing in Queensland between 01 January 2022 and 31 December 2023 inclusive (*n* = 113,555). This study period was chosen to capture two complete RSV seasons of the southern hemisphere [9, 17]. Aligned with Queensland Health RSV notification data practices, consecutive RSV notifications for the same child were counted as discrete infections if they occurred within 180 completed days.

### Variables and data sources

#### Case ascertainment

RSV cases were identified from the Queensland Notifiable Conditions System (NOCS), which records all laboratory-confirmed cases notified by health service providers under the *Public Health Act 2005* [22]. Case ascertainment followed the Australian national surveillance case definition, requiring laboratory confirmation by cell culture, viral antigen detection, seroconversion, or, from 1 July 2023, point-of-care testing resulting in the detection of RSV virus by nucleic acid testing (Supporting Information, box 1) [17].

Patients are asked at the point of testing whether they identify as Aboriginal and/or Torres Strait Islander. This information is uploaded into NOCS, where ‘Indigenous status’ is an identification variable with 4 options: Aboriginal, Torres Strait islander, Both, neither. This variable was then collapsed into a binary 0,1 for analysis 0 = non-Indigenous, 1 = Indigenous.

#### Population estimates

Population estimates for incidence calculations were obtained from 2021 Australian Bureau of Statistics (ABS) census data [23]. For spatial analysis, census population estimates were obtained by single year of age (0-year-olds and 1-year-olds) and postcode area.

#### Community characteristics

Characteristics for each notification were obtained from the NOCS register, including age and residential postcode. Quintiles of socioeconomic disadvantage for each postcode area were assigned based on the Index of Relative Socioeconomic Disadvantage (IRSAD) within the ABS’s Socioeconomic Index for Areas (SEIFA) [24], which ranks postcodes according to relative socioeconomic advantage and disadvantage using indicators such as income, education, employment, occupation, housing, and family structure. Average number of children per family household was extracted from ABS 2021 census ‘All persons QuickStats’ for all postcode areas [25]. Remoteness of residential postcode area was ascertained from the Accessibility/Remoteness Index of Australia (ARIA+) [26]. This index is assigned based on the distance from usual place of residence to health care and other services. Categories include major cities, inner regional, outer regional, remote, and very remote.

#### Climate zone

Climate zones were determined using the Australian Building Code Board (ABCB) climate zone designation established in 2015, which are assigned to each postcode area in Australia [27]. ABCB climate zones are derived from Australian Bureau of Meteorology climate zones, which describe temperature and humidity in both January and July [27, 28]. Four out of the total eight Australian climate zones are present in Queensland. Zone 1: high humidity summer, warm winter (tropical); Zone 2: warm humid summer, mild winter (subtropical); Zone 3: hot dry summer, warm winter (arid/semi-arid); and Zone 5: warm temperate [27].

### Data analyses

Incidence of RSV notifications were calculated per 1,000 total Queensland population, by 5-year age group and year of notification, then by each completed month of age for children aged < 2 years. Monthly age denominators were derived by splitting ABS single-year-of-age population estimates into twelfths. All eligible RSV notifications in Queensland for children aged < 2 years during 1 January 2022–31 December 2023 were included. As the study used a complete population dataset, no formal sample size calculation was required. Epidemic curves were constructed to demonstrate weekly incidence among children, by climate zone of residential postcode and notification year. Covariate selection was informed by the existing evidence base, with crowding, socioeconomic status, and remoteness included due to their established associations with infectious disease incidence. Poisson regression (generalized linear model) was used to calculate the relative risk (RR) of RSV notifications among children by postcode and climate zone (index: zone 1, high humidity summer, warm winter), IRSAD quintile (for each one-quintile reduction in advantage), ARIA designation (for each one-unit increase in remoteness category) and family size (for each one-unit increase in mean number of children per family household). We evaluated random-effect variance and employed a likelihood ratio test comparing models with and without postcode-level random intercepts to confirm the necessity of multilevel specification. Inclusion of postcode-level random effects significantly improved model fit. Model fit was also assessed using Akaike’s information and Bayesian information criterion i.e. lower AIC/BIC indicates a better fitting model. Adjusted RRs (aRRs) were subsequently calculated including all covariates. Results are presented as RRs/aRRs with corresponding 95% confidence intervals (95% CI).

We applied Kulldorff’s spatial scan statistic (Poisson model) to detect high-incidence spatial clusters of observed vs. expected RSV notifications by year. The model included population offsets, calculated as the natural logarithm of the population aged < 2 years within each postcode area. P-values were obtained through Monte Carlo hypothesis testing (99,999 iterations), and clusters deemed as significant where *p* < 0.05.

Data were cleaned and analysed using the statistical software package StataSE v.18, and graphs and figures were constructed using StataSE v.18 (StataCorp LLC, College Station, TX, USA), and Microsoft Excel version 16.0 (Microsoft Corporation, Redmont, WA). Spatial clustering analysis was conducted using SaTScan v10.2.1 and maps developed using ArcGIS v10.8.2. SaTScan outputs do not provide 95% CIs.

### Bias

Analyses were restricted to variables available in the mandatory notification dataset and postcode-level community characteristics. Several relevant demographic and clinical covariates (e.g. daycare attendance, household composition, comorbidities, and vaccination status or coverage) were unavailable, and a degree of residual confounding is therefore likely. Misclassification bias may also be present, as postcode of residence was recorded at time of testing and may not reflect residence at the 2021 census. In addition, census-derived postcode population denominators may contain error due to miscount or privacy-related adjustments, potentially leading to over- or underestimation of RSV incidence in areas with small infant populations. Finally, underascertainment is likely, particularly in remote and socioeconomically disadvantaged communities with reduced access to testing and healthcare services, which may result in underestimated incidence compared with more advantaged or metropolitan areas.

### Ethics approval

Unconditional ethics approval was attained for this project in February 2023 by the Human Research Ethics Committees (HREC) from The University of Queensland and Queensland Health, approval number HREC/2023/MNHA/96,960.

## Results

### Population characteristics

According to the 2021 ABS census, Queensland had a population of approximately 5.14 million [25]. Between 2022−2023 there were 58,019 RSV notifications in Queensland. First Nations status was missing for 25% of all notifications (*n* = 14,647/58,019), with very high missingness in many postcode areas (50–100%). Due to the high overall proportion of missing data and considerable spatial variability in missingness, no further analyses were conducted by First Nations status.

### Incidence of RSV notifications

#### Total Queensland population by 5-year age group

There were 58,019 RSV notifications in Queensland, representing an incidence of 5.7 cases per 1,000 total population in 2022, and 5.6 notifications per 1,000 in 2023. By age group, incidence was highest among young children aged < 5 years, with 49.2 notifications per 1,000 children (Supporting Information, Fig. 1).

**Fig. 1:**
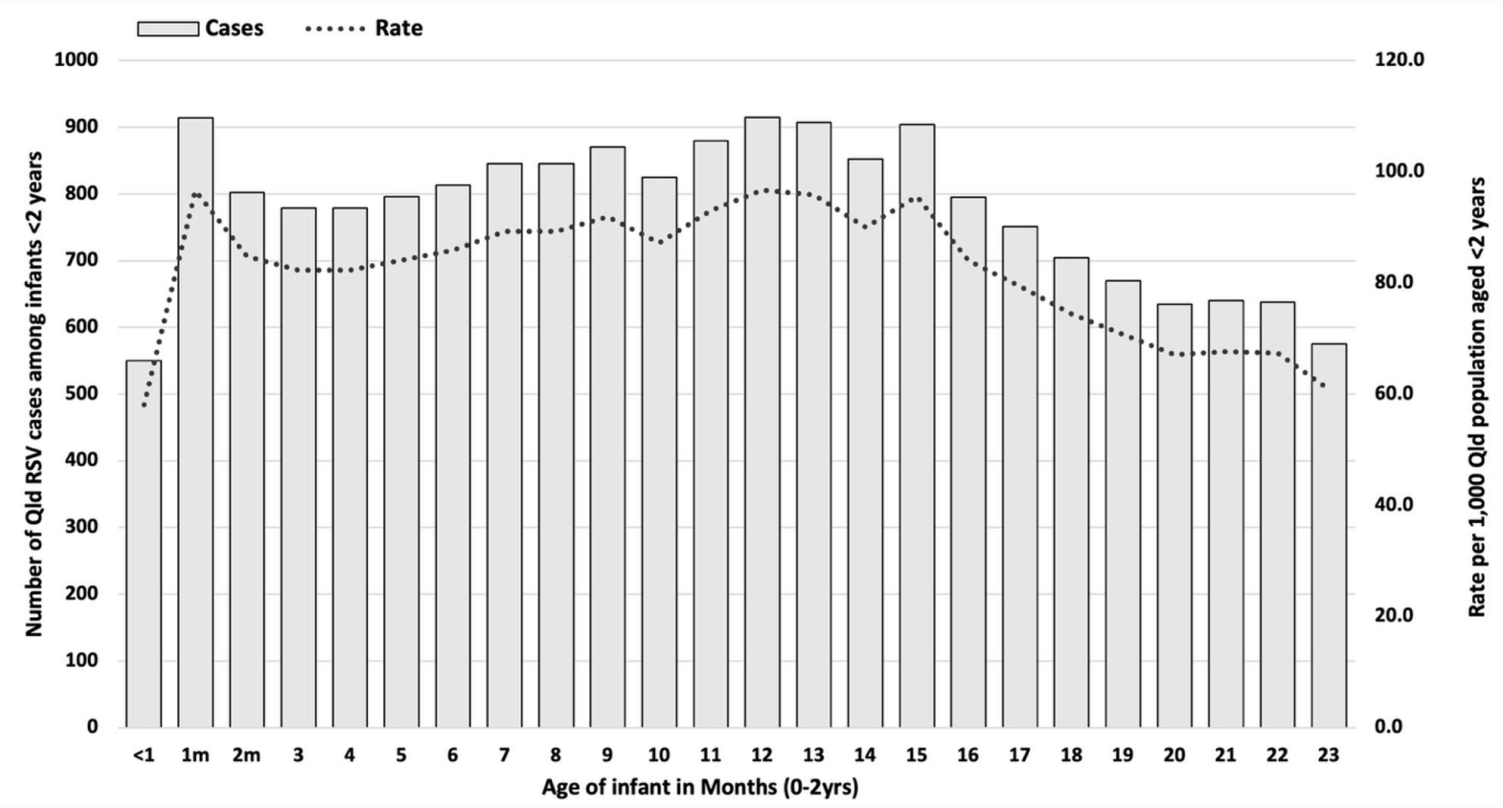
Number and incidence of RSV infections in Queensland children aged < 2 years by month of age, 2022–2023 inclusive

#### Children aged < 2 years

The annual population < 2 years in Queensland in 2022 and 2023 was 113,555. Of the 58,019 total Queensland notifications, 18,683 were among children aged < 2 years (32.2% of all notifications). Of these, 10,160 were male (54.4% of notifications). Overall incidence among children was 82.26 cases per 1,000 (79.73 cases per 1,000 in 2022; 84.80 cases per 1,000 in 2023) (Table 1). Incidence was higher in 2023 compared to 2022 (RR 1.06, 95% CI 1.04−1.09).

**Table 1:** Incidence of Queensland RSV notifications per 1000 children aged < 2 years in 2022–2023, by year and climate zone

Climate zone^a^	Population aged < 2yrs^b^	2022 Notification count, incidence (95% CI)^c^	2023 Notification count, incidence, (95% CI) ^c^	Pooled 2022-2023 Notification count, incidence, (95% CI)^c^
**Zone 1: ** High humidity summer, warm winter (tropical)	11 769	*810*, 68.88 (64.25–73.40)	*938*, 79.70 (74.81–84.59)	*1 748*, 74.26 (70.91–77.61)
**Zone 2: ** Warm humid summer, mild winter (subtropical)	91 734	*7 325*, 79.85 (78.10–81.61.10.61)	*7 884*, 85.94 (84.13–87.80)	*15 209*, 82.90 (81.64–84.20)
**Zone 3: ** Hot dry summer, warm winter (arid/semi-arid)	4 638	*381*, 82.15 (74.25–90.10)	*251*, 54.12 (47.61–60.63)	*632*, 68.13 (63.01–73.26)
**Zone 5: ** Warm temperate	5 414	*538*, 99.37 (91.40–107.34.40.34)	*556*, 102.70 (94.61–110.78.61.78)	*1 094*, 101.03 (95.36–106.71.36.71)
**Totals**	113 555	*9 054*, 79.73 (78.16–81.31)	*9 629*, 84.80 (83.18–86.42)	*18 683*, 82.26 (81.13–83.39)

^a^Australian Building Codes Board climate zone, by postcode area

^b^Population denominators derived from Australian Bureau of Statistics 2021 Census single-year age estimates for Queensland, by postcode area

^c^Incidence per 1,000 children aged < 2yrs, with 95% confidence interval (Wald)

By 1-month age group, incidence of RSV notifications was consistently high from 1 to 15 months of age (range 82.3−96.7 per 1,000) (Fig. 1; Supporting Information, Table 1). Highest incidence occurred among 1-month-olds (96.6 per 1,000) and 12-month-olds (96.7 per 1,000), where “1-month-old” and “12-month-old” reflect the exact age at notification. There was a downward trend in incidence after 15 months of age (Fig. 1).

### RSV incidence by climate zone

The highest crude incidence among children aged < 2 years occurred in warm temperate areas (zone 5, 101.0 per 1 000 children), and this was consistently observed across 2022 and 2023 (Table 1). In the adjusted model (Table 2), arid/semi-arid (zone 3, aRR 1.18, 95% CI 1.00−1.38) and warm temperate areas (zone 5, aRR 1.26, 95% CI 1.13−1.41) had higher overall incidence compared to tropical areas (zone 1). There was no difference in incidence detected between tropical and subtropical areas (zone 2, aRR 1.02, 95% CI 0.90−1.15) (Table 2). Climate zone 4 is not present in Queensland.

**Table 2:** Poisson regression models investigating community-level determinants of RSV incidence by Australian postcode area in 2022–2023

	Observations	RR^1^ (95% CI)	Adjusted RR^a, b^ (95% CI)
Climate zone			
Zone 1: High humidity summer, warm winter (tropical)	51	Reference category	Reference category
Zone 2: Warm humid summer, mild winter (subtropical)	228	1.25 (1.15–1.36)*	1.02 (0.90–1.15)
Zone 3: Hot dry summer, warm winter (arid/semi-arid)	53	1.23 (1.04–1.47)*	1.18 (1.00–1.38)*
Zone 5: Warm temperate	36	1.46 (1.32–1.61)*	1.26 (1.13–1.41)*
Average number of children per family household ^c^	**363**	1.24 (0.99–1.56)	1.39 (1.13–1.72)*
Socioeconomic disadvantage (IRSAD quintile)^d^	**363**	1.02 (1.00–1.04)*	1.00 (0.98–1.01)
Remoteness (ARIA category)^e^	**363**	0.91 (0.88–0.94)*	0.89 (0.87–0.92)*

*Statistically significant results (*p* < 0.05 level)

^a^Risk Ratio; obtained using Poisson regression

^b^Adjusted models include climate zone, average number of children per family household, socioeconomic disadvantage (IRSAD quintile) and remoteness (ARIA category), by Australian postcode area

^c^RR for each 1-unit increase in mean number of children per family household

^c^RR for each 1-unit increase in socioeconomic disadvantage of postcode area

^d^RR for each 1-unit increase in remoteness of postcode area

### Variation in timing of RSV season by climate zone

**Fig. 2:**
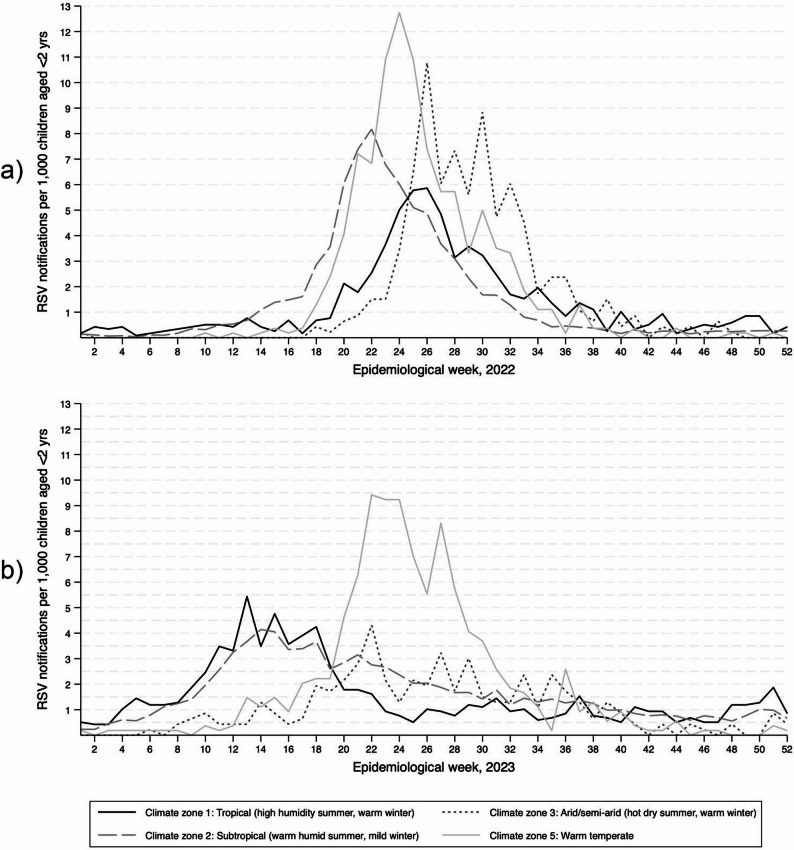
Incidence of RSV notifications in Queensland per 1,000 children aged < 2 years, by year, epidemiological week, and climate zone of residence Supporting Information

In 2022, incidence of RSV notifications by epidemiological week showed a monophasic pattern typical of a southern hemisphere annual late-autumn to mid-winter peak (May − July). In 2023, most notifications occurred during autumn (March − May). Weekly RSV notifications for both years showed distinct differences in their distribution between climate zones. In 2022, there was an apparent one-month difference between the peak of RSV notifications for climate zones 1 and 2 and 2 and 3, and multiple peaks throughout the year for zone 3 (Fig. 2a; Supporting Information, Table 3). In 2023, there was year-round elevated baseline RSV notifications for climate zones 1 and 2 with peaks occurring between March − May. Multiple peaks of notifications occurred in climate zones 3 and 5, with bimodal peaks in zone 5 during the winter months. There was a difference of approximately two months between peaks for climate zones 1, 3 and 5 (Fig. 2b; Supporting Information, Table 3).

### Spatial clustering of notifications by postcode and year

**Fig. 3:**
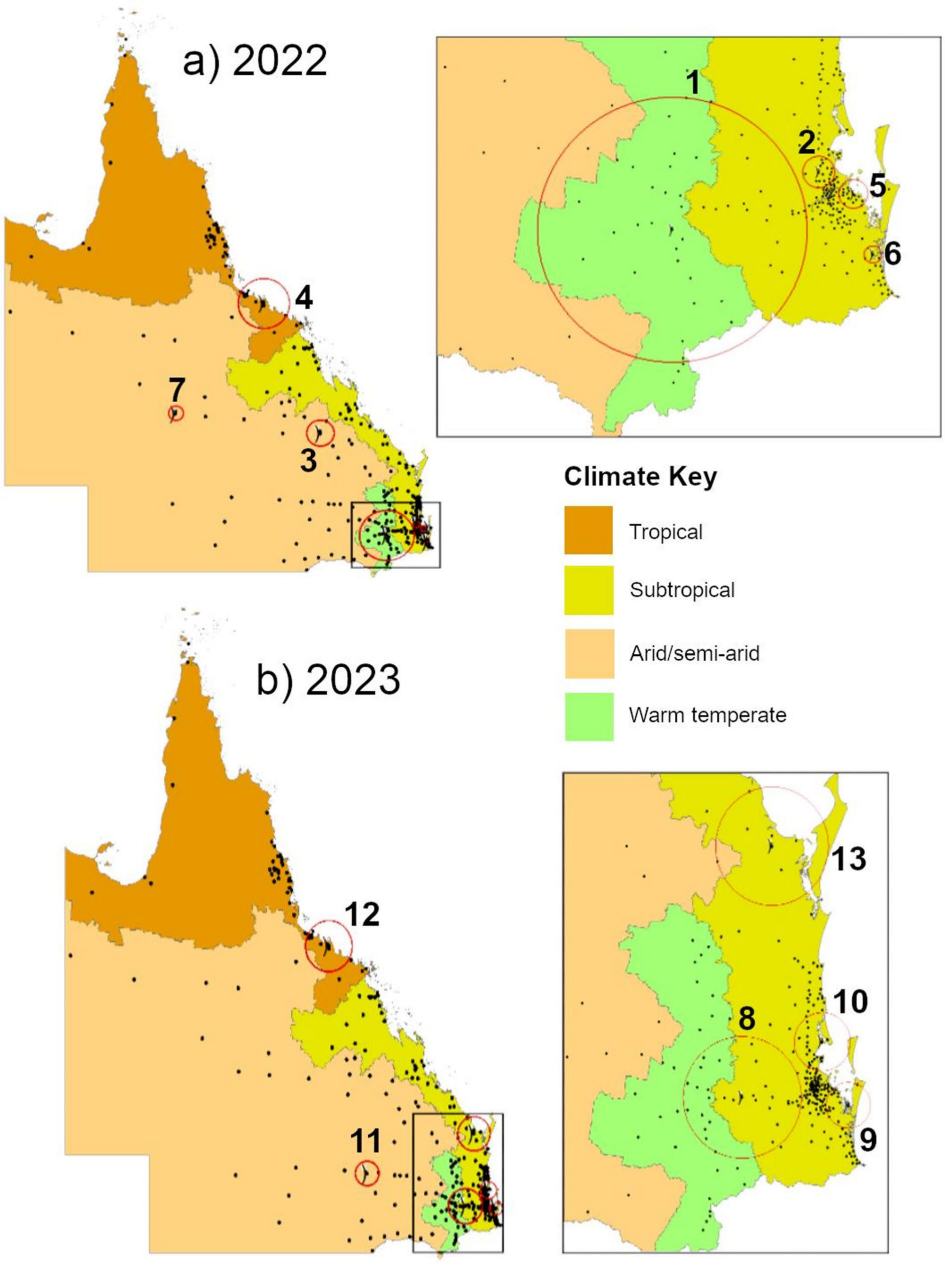
Cluster analysis of Queensland RSV notifications per 1,000 children aged < 2 years by year and residential postcode, with ABCB climate zone boundaries. Cluster key (**a**)2022: 1 – Darling Downs region (Toowoomba), 2 – Samford (Greater Brisbane), 3 – Aboriginal Shire of Woorabinda, 4 – Townsville and surrounds, 5– Redland (Greater Brisbane), 6 – Gold Coast, 7 – Longreach.** b **2023: 8 – Darling Downs (Gatton), 9 – Logan (Greater Brisbane), 10 – Moreton Bay (Greater Brisbane/Sunshine Coast), 11 – Roma, 12 – Townsville and surrounds, 13 – Hervey Bay/Maryborough

Incidence of RSV notifications differed between residential postcode areas and years (Supporting Information, Fig. 3). Compared to the rest of Queensland, high-incidence clusters occurred in both years within the major population centres of Greater Brisbane (multiple clusters: localities and RRs in Supporting information, Table 2), Townsville and surrounds (2022: RR 1.26, *p* < 0.01; 2023: RR 1.16, *p* < 0.01), Darling Downs (2022: RR 1.39, *p* < 0.01; 2023: RR 1.48, *p* < 0.01), and the Gold Coast in 2022 (RR 1.20, *p* < 0.01) (Fig. 3; Supporting Information, Table 2). In 2022, regional clusters were identified in the Aboriginal Shire of Woorabinda (RR 5.20, *p* < 0.01) (where approximately 90% of residents identify as Aboriginal and/or Torres Strait Islander) [29], and the Longreach area (RR 3.22, *p* = 0.04) (Fig. 3a, Supporting Information, Table 2). In 2023, regional clusters were identified in the Hervey Bay-Maryborough (RR 1.28, *p* = 0.01) and Roma areas (RR 2.36, *p* < 0.01) (Fig. 3b, Supporting Information, Table 2).

### Community characteristics and RSV incidence

In the unadjusted model, there was weak evidence of increased incidence for each one-quintile increase in socioeconomic disadvantage (RR 1.02, 95% CI 1.00−1.04). This association was no longer statistically significant following adjustment (aRR 1.00, 95% CI 0.98−1.01) (Table 2). In contrast, the incidence of RSV notifications decreased significantly with each one-quintile increase in remoteness of postcode area (RR 0.91, 95% CI 0.88−0.94; aRR 0.89, 95% CI 0.84−0.94). In addition, there was a 39% increase in RSV incidence observed for each additional child in the mean number of children per family household by postcode (aRR 1.39, 95% CI 1.13−1.72) (Table 2).

## Discussion

Among children aged < 2 years, incidence of RSV was persistently high from 1 to 15 months of age. Highest incidence was recorded among 1-month-olds and 12-month-olds, the latter of whom are currently ineligible for protection with nirsevimab under the RSV-MIPP unless they have additional risk factors for severe disease. We identified high-incidence spatial clusters of RSV notifications in both major population centres and regional areas and found that remoteness of residence and number of children per family household may be important community-level determinants of RSV incidence. The role of community-level socioeconomic disadvantage in determining incidence was less clear, possibly due to unmeasured confounding from socioeconomic differences in utilisation of healthcare, childcare, and other services. RSV incidence varied between Queensland’s climate zones, with greater incidence recorded in lower humidity areas with a more distinct winter season. Timing and magnitude of RSV epidemics among children also differed between Queensland’s climate zones, highlighting differences in key risk periods for RSV transmission across the state.

Given the frequency of severe outcomes among infants aged < 6 months [1, 2] and compelling evidence of a causal association with the onset of childhood asthma following RSV infections in the first year of life [2, 30], high incidence of RSV among infants represents a major public health issue. The RSV-MIPP is likely to reduce incidence and severity among Australia’s youngest infants, however we observed persistently high RSV incidence from birth until 15 months of age. Maternal RSV vaccination and nirsevimab administered at birth have each been demonstrated to protect infants until approximately 6 months of age [31, 32]. Older infants > 6 months of age are unlikely to be adequately protected by current interventions. Options to extend the duration of protection against RSV into early childhood should be explored.

Family size, household crowding, and RSV transmission dynamics are important considerations in future program planning. Household transmission between siblings is a major driver of RSV incidence, with children who are exposed to RSV at school or daycare subsequently spreading infections to other children in their home [5, 33]. In light of this, nirsevimab and maternal RSV vaccination may be particularly cost-effective when used to protect infants who live with older preschool or school-aged siblings for whom RSV immunisation is not yet available [13]. Childcare centres are an effective setting to raise awareness of RSV and its prevention among parents.

The effect of community-level socioeconomic disadvantage on RSV incidence among children was not statistically significant in the adjusted model, and increasing remoteness appeared to be associated with lower RSV incidence. Considering that remote and disadvantaged communities in Australia are often burdened by established risk factors for RSV transmission such as household crowding [34], these results should be interpreted cautiously. Our findings may be explained by lower rates of RSV testing in more socially disadvantaged or remote regions, smaller populations in remote areas, or residual effects of the COVID-19 pandemic. It is also plausible that remoteness may be associated with decreased incidence of RSV among children due to lower and/or later initiation of attendance at formal childcare services in rural and remote areas of Queensland, where service availability is often limited [35]. Similarly, the high cost of centre-based childcare in Australia is associated with reduced attendance among children from socioeconomically disadvantaged households [35]. Future studies may elucidate whether access to local childcare services is an independent spatial determinant of RSV notifications. Despite our observations, it is well established that socioeconomically disadvantaged and remote residents experience significant cost, availability, distance, and cultural barriers to accessing health care services in Australia [36]. Prioritising equity in new RSV prevention programs is critical to ensure optimal coverage among all eligible infants.

Queensland’s tropical and subtropical zones demonstrated year-round circulation of RSV with lower-incidence peaks, which occurred earlier in 2023 compared to other zones. Previous studies have found peaks occurring during periods of high rainfall in North Queensland and other tropical regions [7, 9]; with lower overall incidence thought to be determined by a combination of consistent high humidity and lack of a distinctly cooler winter season [7]. Findings were similar for subtropical areas, albeit with a more distinct seasonal peak in 2022 than observed in the tropics. Whilst overall incidence was lower in the tropical and subtropical zones compared to other parts of Queensland, significant clusters were still identified in several densely populated areas within these zones.

Epidemics in warm temperate (zone 5) and arid/semi-arid (zone 3) regions with distinct winter seasons were more discrete, with consistent high-incidence seasonal peaks observed in zone 5 across 2022−2023. Incidence in the arid/semi-arid interior regions of Queensland was high in 2022, but this was not repeated in 2023. There is some evidence that RSV outbreaks may occur biennially in some non-tropical regions of Australia [8], and future years of RSV notification data will clarify whether this is true for Queensland’s dry interior. Understanding these patterns will be necessary for targeted vaccination program planning and rollout.

### Limitations

Notification data inherently underestimates true RSV incidence, as testing is prerequisite to notification. Evidence from Western Australia suggests that the true incidence of RSV among children aged < 5 years may be 30−57% higher than captured through RSV notifications [37], and this estimate may be conservative. Incidence in our study is therefore likely to be underestimated, particularly among populations with lower rates of testing due to poorer access to health services, milder symptoms, or in lower-risk age groups.

Due to high missingness for First Nations status with considerable spatial variation, we were unable to reliably assess spatial incidence of RSV notifications among First Nations children. Our study did however identify a major RSV cluster among children in the Woorabinda Shire, where 91.6% of residents identify as a First Nations person [29]. Data sources with greater completeness for this important variable and improved capture of First Nations status at the time of RSV testing are necessary to understand community-level determinants of RSV incidence among First Nations children.

It was not possible to identify notifications among siblings from the same household in our unlinked dataset. Mean number of children per family household in each postcode area was examined as a community-level determinant of RSV incidence, however infants with RSV notifications may have lived in households with an above or below average number of children compared to their postcode area. This is also true of IRSAD quintile, as individual infants may have greater or lesser degrees of socioeconomic advantage compared to their area of residence.

Whilst our findings are relevant for Queensland, further research is required to ascertain whether these findings are generalisable to other jurisdictions.

## Conclusion

The introduction of the RSV-MIPP is likely to reduce incidence of RSV infections and severe outcomes among younger infants, however older children are unlikely to be adequately protected. Protective strategies for these children warrant further attention. We found higher incidence of RSV notifications in 2022−2023 in areas with larger family sizes, and in areas with distinctly cooler and lower humidity winters. Further research is needed to establish a better understanding of RSV seasonality in arid zones. Lower RSV incidence observed in remote areas may be attributable to lower rates of testing, or limited access to childcare services. Community-level determinants of RSV incidence should be considered in RSV-MIPP evaluation and planning.

## Supplementary Information

Supplementary Material 1.

## Data Availability

ABS 2021 census data utilised in this study can be accessed at the following URL: [https://www.abs.gov.au/census/find-census-data/datapacks]The Australian Building Codes Board climate zone mapping dataset can be accessed at the following URL: [https://data.gov.au/data/dataset/australian-climate-zone-map]Deidentified individual patient-level RSV notification data utilised in this study cannot be made publicly available for ethical and privacy reasons. Requests to the corresponding author for the data will be considered on a case-by-case basis.

## Abbreviations

ABCB: The Australian Building Code Board
ABS: Australian Bureau of Statistics
ARIA+: Accessibility/Remoteness Index of Australia
aRRs: Adjusted relative risks
CI: Confidence interval
EL1: Emerging Leadership
HREC: Human Research Ethics Committees
IRSAD: Index of Relative Socioeconomic Disadvantage
NIP: National Immunisation Program
NOCS: Queensland Notifiable Conditions System
RR: Relative risk
RSV-MIPP: Respiratory Syncytial Virus Mother and Infant Protection Program
RSV: Respiratory Syncytial Virus
SEIFA: Socioeconomic Index for Areas
TGA: Therapeutic Goods Administration
