# Supplementary material for: A retrospective cohort study and spatial analysis of climate and community-level determinants of respiratory syncytial virus notifications among Queensland infants, prior to the introduction of the RSV mother and infant protection program (RSV-MIPP) immunisation initiative
Source: BMC Public Health. 2026 Jan 22;26:615. doi: 10.1186/s12889-026-26288-6 (PMC12910892; doi:10.1186/s12889-026-26288-6)
Supplement: Supplementary file 1 — Supplementary Material 1. [file 12889_2026_26288_MOESM1_ESM.docx]

# Supporting Information

| ***Confirmed case*** *–* Definitive laboratory evidence only |
| --- |
| *Laboratory definitive evidence:*   1. Isolation of respiratory syncytial virus by a cell culture   OR   1. Detection of respiratory syncytial virus by nucleic acid testing   OR   1. Detection of respiratory syncytial virus antigen   OR   1. Seroconversion, or a significant increase in antibody level such as a fourfold or greater rise in titre, to respiratory syncytial virus between paired sera of immunoglobulin G (IgG) or total antibody |

**Supporting information, box 1:** Respiratory syncytial virus Australian national notifiable diseases case definition, from: <https://www.health.gov.au/resources/publications/respiratory-syncytial-virus-surveillance-case-definition> (viewed April 2025).


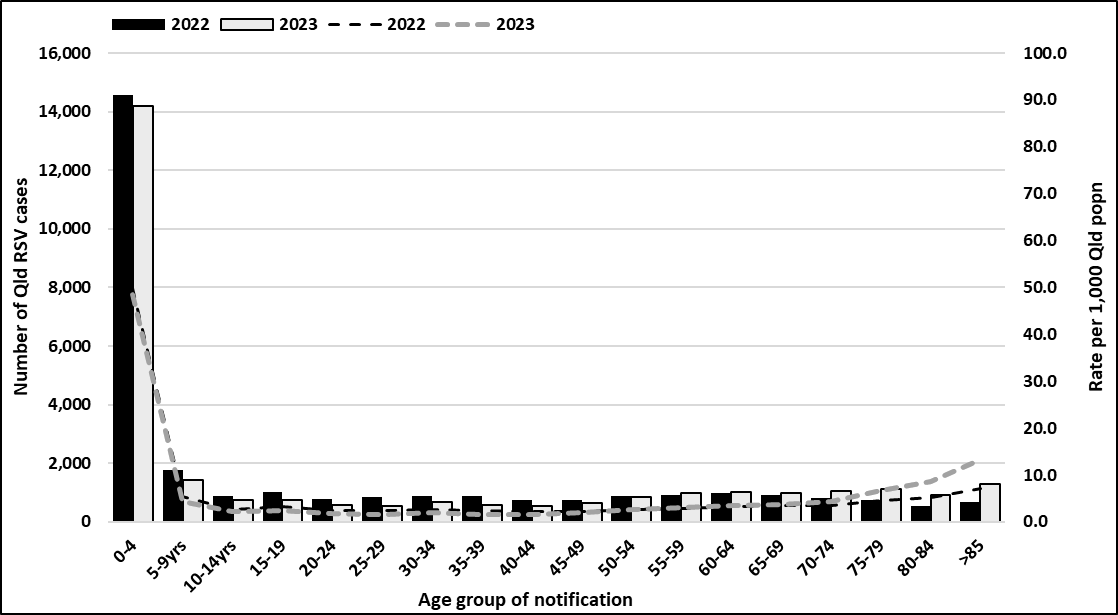


**Supporting information, figure 1**. Number and incidence of RSV notifications in Queensland 2022–2023 inclusive.

**Supporting information, table 1.** Number and incidence of RSV infections per 1,000 children aged <2 years in Queensland by completed month of age, 2022–2023 inclusive.

| **Age (months)** | **RSV notifications (count)** | **Incidence (95% CI)^a^** |
| --- | --- | --- |
| 0 | 550 | 58.12 (53.41-62.84) |
| 1 | 914 | 96.59 (90.64-102.54) |
| 2 | 802 | 84.75 (79.14-90.36) |
| 3 | 779 | 82.32 (76.78-87.86) |
| 4 | 779 | 82.32 (76.78-87.86) |
| 5 | 796 | 84.12 (78.53-89.71) |
| 6 | 813 | 85.91 (80.27-91.56) |
| 7 | 845 | 89.30 (83.55-95.04) |
| 8 | 845 | 89.30 (83.55-95.04) |
| 9 | 870 | 91.94 (86.12-97.76) |
| 10 | 825 | 87.18 (81.50-92.87) |
| 11 | 880 | 93.00 (87.14-98.85) |
| 12 | 915 | 96.69 (90.74-102.65) |
| 13 | 907 | 95.85 (89.92-101.78) |
| 14 | 852 | 90.04 (84.27-95.80) |
| 15 | 904 | 95.53 (89.61-101.45) |
| 16 | 795 | 84.01 (78.42-89.60) |
| 17 | 751 | 79.36 (73.92-84.81) |
| 18 | 704 | 74.40 (69.11-79.68) |
| 19 | 670 | 70.80 (65.63-75.97) |
| 20 | 635 | 67.10 (62.06-72.15) |
| 21 | 640 | 67.63 (62.57-72.69) |
| 22 | 638 | 67.42 (62.37-72.47) |
| 23 | 575 | 60.76 (55.95-65.58) |
| TOTAL | 18,683 | **82.26 (81.13-83.39)** |

^a^Population denominators derived from Australian Bureau of Statistics 2021 Census single-year age estimates for Queensland
^b^Incidence per 1,000 children aged <2yrs, with 95% confidence interval (Wald)


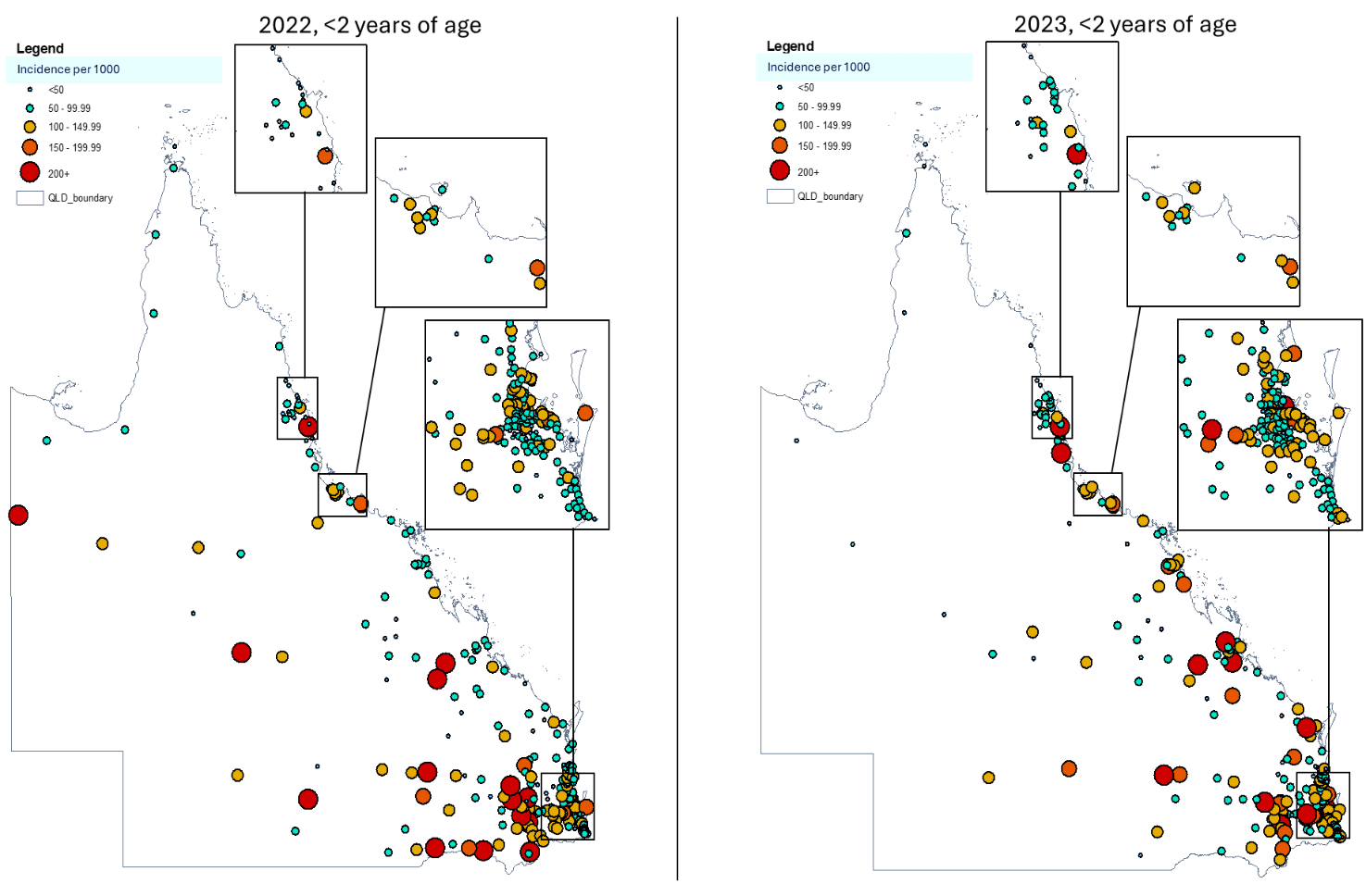


**Supporting information, figure 2**. Incidence of RSV notifications by postcode (location based on population weighted centroid) in Queensland per 1,000 children aged <2 years

**Supporting information, table 2.** Spatial cluster analysis of Queensland RSV infections among children aged <2 years, 2022–2023

| **Location of spatial clusters,  by year** | **Cases (n)** | **O/E^a^ ratio** | **Relative risk (p-value)** | **Climate zone^b^** |
| --- | --- | --- | --- | --- |
| **2022** |  |  |  |  |
| 1: Darling Downs region (Toowoomba) | 1189 | 1.34 | 1.39 (<0.001) | 2 & 5 |
| 2: Samford (Greater Brisbane) | 1060 | 1.25 | 1.28 (<0.001) | 2 |
| 3: Aboriginal Shire of Woorabinda | 24 | 5.19 | 5.20 (<0.001) | 3 |
| 4: Townsville and surrounds | 502 | 1.24 | 1.26 (<0.001) | 1 |
| 5: Redland (Greater Brisbane) | 496 | 1.17 | 1.18 (<0.001) | 2 |
| 6: Gold Coast | 410 | 1.19 | 1.20 (0.008) | 2 |
| 7: Longreach | 19 | 3.21 | 3.22 (0.039) | 3 |
| **2023** |  |  |  |  |
| 8: Darling Downs (Gatton) | 1223 | 1.42 | 1.48 (<0.001) | 2 & 5 |
| 9: Logan (Greater Brisbane) | 755 | 1.33 | 1.36 (<0.001) | 2 |
| 10: Moreton Bay (Greater Brisbane/Sunshine Coast) | 1016 | 1.13 | 1.14 (<0.001) | 2 |
| 11: Roma | 56 | 2.35 | 2.36 (<0.001) | 3 |
| 12: Townsville and surrounds | 450 | 1.15 | 1.16 (0.003) | 1 |
| 13: Hervey Bay-Maryborough | 357 | 1.27 | 1.28 (0.009) | 2 |

^a^Observed/Expected ratio

^b^Climate zone key:
1 = High humidity summer, warm winter (tropical)
2 = Warm humid summer, mild winter (subtropical)
3 = Hot dry summer, warm winter (semi-arid/arid)
5 = Warm temperate

**Supporting information, table 3.** Incidence of RSV notifications in Queensland per 1,000 children aged <2 years, by year, epidemiological week and climate zone of residence

| **Epidemiological week, by year** | **Incidence^a^, zone 1 (tropical)** | **Incidence^a^, zone 2 (subtropical)** | **Incidence^a^, zone 3 (arid/semi-arid)** | **Incidence^a^, zone 5 (warm temperate)** |
| --- | --- | --- | --- | --- |
| **2022** |  |  |  |  |
| 1 | 0.17 | 0.14 | 0.22 | 0.00 |
| 2 | 0.42 | 0.11 | 0.00 | 0.00 |
| 3 | 0.34 | 0.07 | 0.00 | 0.00 |
| 4 | 0.42 | 0.08 | 0.00 | 0.00 |
| 5 | 0.08 | 0.03 | 0.00 | 0.00 |
| 6 | 0.17 | 0.11 | 0.00 | 0.00 |
| 7 | 0.00 | 0.10 | 0.00 | 0.00 |
| 8 | 0.34 | 0.17 | 0.00 | 0.00 |
| 9 | 0.42 | 0.35 | 0.00 | 0.00 |
| 10 | 0.51 | 0.32 | 0.00 | 0.18 |
| 11 | 0.51 | 0.50 | 0.00 | 0.00 |
| 12 | 0.42 | 0.53 | 0.00 | 0.18 |
| 13 | 0.76 | 0.69 | 0.00 | 0.00 |
| 14 | 0.42 | 1.09 | 0.00 | 0.18 |
| 15 | 0.25 | 1.38 | 0.00 | 0.37 |
| 16 | 0.68 | 1.48 | 0.00 | 0.18 |
| 17 | 0.17 | 1.61 | 0.00 | 0.37 |
| 18 | 0.68 | 2.86 | 0.43 | 1.29 |
| 19 | 0.76 | 3.56 | 0.22 | 2.40 |
| 20 | 2.12 | 6.05 | 0.65 | 4.06 |
| 21 | 1.78 | 7.38 | 0.86 | 7.20 |
| 22 | 2.55 | 8.18 | 1.51 | 6.83 |
| 23 | 3.65 | 6.78 | 1.51 | 10.90 |
| 24 | 5.01 | 6.02 | 3.45 | 12.74 |
| 25 | 5.78 | 5.10 | 6.47 | 10.90 |
| 26 | 5.86 | 4.87 | 10.78 | 7.39 |
| 27 | 4.84 | 3.67 | 6.04 | 5.73 |
| 28 | 3.14 | 3.10 | 7.33 | 5.73 |
| 29 | 3.57 | 2.33 | 5.61 | 3.32 |
| 30 | 3.23 | 1.68 | 8.84 | 4.99 |
| 31 | 2.46 | 1.67 | 4.74 | 3.51 |
| 32 | 1.70 | 1.28 | 6.04 | 3.32 |
| 33 | 1.53 | 0.81 | 4.53 | 1.85 |
| 34 | 1.95 | 0.69 | 1.72 | 1.11 |
| 35 | 1.36 | 0.43 | 2.37 | 1.11 |
| 36 | 0.85 | 0.46 | 2.37 | 0.18 |
| 37 | 1.36 | 0.41 | 1.08 | 1.29 |
| 38 | 1.10 | 0.38 | 0.65 | 0.37 |
| 39 | 0.25 | 0.27 | 1.51 | 0.37 |
| 40 | 1.02 | 0.16 | 0.43 | 0.00 |
| 41 | 0.34 | 0.31 | 0.86 | 0.37 |
| 42 | 0.51 | 0.19 | 0.00 | 0.00 |
| 43 | 0.93 | 0.26 | 0.43 | 0.00 |
| 44 | 0.17 | 0.25 | 0.22 | 0.37 |
| 45 | 0.34 | 0.15 | 0.43 | 0.00 |
| 46 | 0.51 | 0.23 | 0.00 | 0.00 |
| 47 | 0.42 | 0.26 | 0.65 | 0.00 |
| 48 | 0.59 | 0.24 | 0.22 | 0.18 |
| 49 | 0.85 | 0.26 | 0.00 | 0.18 |
| 50 | 0.85 | 0.27 | 0.00 | 0.00 |
| 51 | 0.17 | 0.27 | 0.00 | 0.18 |
| 52 | 0.42 | 0.26 | 0.00 | 0.00 |
| **2023** |  |  |  |  |
| 1 | 0.51 | 0.23 | 0.00 | 0.18 |
| 2 | 0.42 | 0.23 | 0.00 | 0.00 |
| 3 | 0.42 | 0.46 | 0.00 | 0.18 |
| 4 | 1.02 | 0.60 | 0.00 | 0.18 |
| 5 | 1.44 | 0.57 | 0.00 | 0.18 |
| 6 | 1.19 | 0.78 | 0.22 | 0.18 |
| 7 | 1.19 | 1.11 | 0.00 | 0.18 |
| 8 | 1.27 | 1.24 | 0.43 | 0.18 |
| 9 | 1.87 | 1.41 | 0.65 | 0.00 |
| 10 | 2.46 | 1.96 | 0.86 | 0.37 |
| 11 | 3.48 | 2.56 | 0.43 | 0.18 |
| 12 | 3.31 | 3.25 | 0.43 | 0.37 |
| 13 | 5.44 | 3.67 | 0.43 | 1.48 |
| 14 | 3.48 | 4.14 | 1.29 | 1.11 |
| 15 | 4.76 | 4.06 | 0.86 | 1.48 |
| 16 | 3.57 | 3.36 | 0.43 | 0.92 |
| 17 | 3.91 | 3.39 | 0.65 | 2.03 |
| 18 | 4.25 | 3.66 | 1.94 | 2.22 |
| 19 | 2.72 | 2.59 | 1.72 | 2.22 |
| 20 | 1.78 | 2.85 | 2.16 | 4.62 |
| 21 | 1.78 | 3.15 | 2.80 | 6.28 |
| 22 | 1.61 | 2.76 | 4.31 | 9.42 |
| 23 | 0.93 | 2.67 | 2.16 | 9.24 |
| 24 | 0.76 | 2.37 | 1.29 | 9.24 |
| 25 | 0.51 | 2.04 | 2.16 | 7.02 |
| 26 | 1.02 | 2.04 | 1.94 | 5.54 |
| 27 | 0.93 | 1.87 | 3.23 | 8.31 |
| 28 | 0.76 | 1.67 | 1.72 | 5.73 |
| 29 | 1.19 | 1.68 | 3.02 | 4.06 |
| 30 | 1.10 | 1.42 | 1.51 | 3.69 |
| 31 | 1.44 | 1.78 | 1.29 | 2.59 |
| 32 | 0.93 | 1.19 | 1.29 | 1.85 |
| 33 | 1.02 | 1.45 | 2.37 | 1.66 |
| 34 | 0.59 | 1.31 | 1.08 | 1.11 |
| 35 | 0.68 | 1.42 | 2.37 | 0.18 |
| 36 | 0.85 | 1.26 | 1.72 | 2.59 |
| 37 | 1.53 | 1.36 | 1.29 | 0.92 |
| 38 | 0.76 | 1.24 | 0.65 | 1.29 |
| 39 | 0.68 | 0.98 | 1.29 | 0.55 |
| 40 | 0.51 | 0.99 | 0.86 | 0.92 |
| 41 | 1.10 | 0.85 | 0.43 | 0.37 |
| 42 | 0.93 | 0.76 | 0.00 | 0.18 |
| 43 | 0.93 | 0.80 | 0.43 | 0.18 |
| 44 | 0.51 | 0.75 | 0.00 | 0.55 |
| 45 | 0.68 | 0.59 | 0.43 | 0.00 |
| 46 | 0.51 | 0.76 | 0.22 | 0.18 |
| 47 | 0.51 | 0.69 | 0.00 | 0.18 |
| 48 | 1.19 | 0.56 | 0.43 | 0.00 |
| 49 | 1.19 | 0.76 | 0.00 | 0.00 |
| 50 | 1.27 | 1.01 | 0.00 | 0.00 |
| 51 | 1.87 | 0.98 | 0.86 | 0.37 |
| 52 | 0.85 | 0.66 | 0.43 | 0.18 |

^a^Incidence per 1,000 children
